# Obstructive Endometrial Polyp: A Case Report

**DOI:** 10.7759/cureus.5385

**Published:** 2019-08-13

**Authors:** Aldo Javier Vázquez Mézquita, Alan Jossimar Zavala Vargas, Jorge Luis Vela Cantorán, Yeni Fernández de Lara Barrera

**Affiliations:** 1 Radiology and Molecular Imaging, The American British Cowdray Medical Center, Mexico City, MEX; 2 Gynecology, Instituto Nacional De Perinatología, Mexico City, MEX

**Keywords:** endometrial polyp, obstructive, hysterosalpingography, transvaginal ultrasound, mri

## Abstract

Endometrial polyps are a common cause of abnormal vaginal bleeding and infertility, which can be identified with different imaging methods. A lack of distention of the endometrial cavity is not a common presentation of endometrial polyps and is associated with endometrial carcinoma. In this article, we present a case of a 30-year-old patient with previous history of infertility and abnormal vaginal bleeding. During a hysterosalpingography (HSG), we were not able to distend the endometrial cavity. Therefore, we performed a transvaginal ultrasound (TVUS) and a pelvic magnetic resonance study, which showed an obstructive endometrial polyp adjacent to the internal cervical os. This structure was successfully removed through hysteroscopy by the gynecology department.

## Introduction

Endometrial polyps are a common cause of abnormal vaginal bleeding affecting women in premenopause and postmenopause. These endometrial-dependent masses arise secondary to estrogen overstimulation, which is responsible for the cyclical endometrial growth. Complications of endometrial polyps not related to malignancy include recurrent miscarriages and abnormal uterine bleeding [1-3]. The risk of malignant transformation of these lesions is estimated between 2-3% [1]. Diagnostic methods such as saline infusion sonography (SIS) and magnetic resonance (MRI) are some examples of imaging studies offered to patients with abnormal vaginal bleeding [4]. In this article, we report an unusual presentation of an endometrial polyp that obstructed the endometrial cavity in a woman of childbearing age with recurrent miscarriages.

## Case presentation

**Figure 1:**
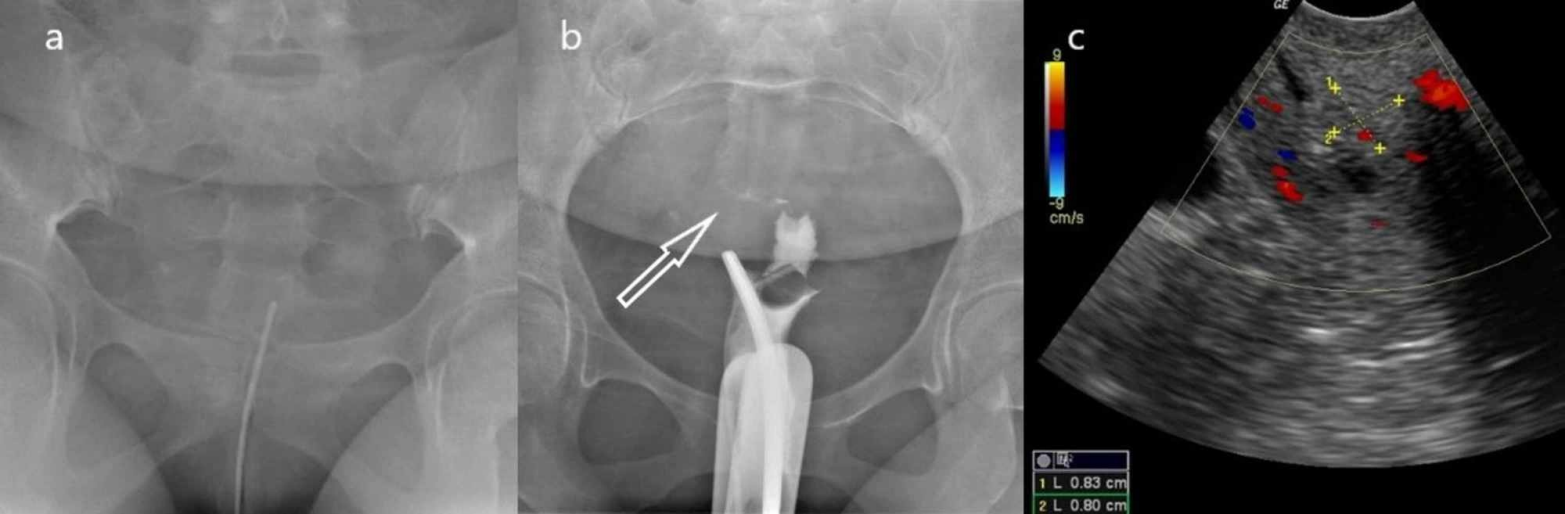
Hysterosalpingography and transvaginal ultrasound images The first image (a) corresponds to the pelvis previous to contrast material injection through the cervical catheter. In the second image (b), total visualization of the cervix is appreciated with a lack of opacification of the endometrial cavity and uterine tubes; the white arrow signals a filiform flow of contrast material in the poorly-distended endometrial cavity. In a transvaginal ultrasound image (c), a round, echogenic, endometrial-dependent structure is observed adjacent to the internal cervical os.

A 30-year-old female patient was sent to our radiology department because of chronic abnormal vaginal bleeding and infertility. The patient denied any pre-existing conditions and only mentioned a long history of miscarriages. She mentioned no other symptoms or complaints. We performed a hysterosalpingography (HSG) in which hydrosoluble iodinated contrast material was injected through a transcervical catheter; it did not reach the endometrial cavity. After contrast material administration, there was a minimal filiform transit of it, as well as a lack of distention of the endometrial cavity. Both of the uterine tubes were not opacified. We decided to perform a transvaginal ultrasound (TVUS), which revealed a round endometrial-dependent structure localized next to the internal cervical os. Images of the HSG and TVUS are shown in Figure 1.

**Figure 2:**
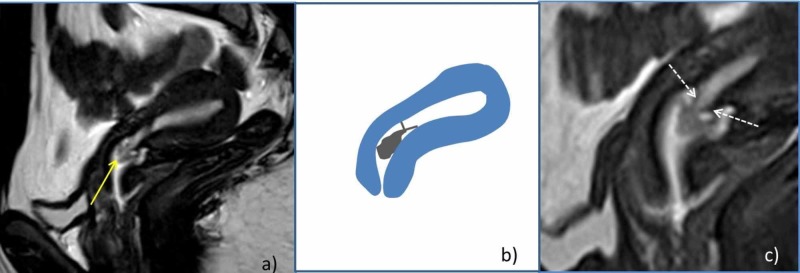
Pelvic magnetic resonance images T2-weighted sagittal images (a, c), the yellow arrow shows an endometrial heterogeneous lesion adjacent to the internal cervical os, which corresponds to an endometrial polyp. The white arrows in Figure 2c signal two thin band-like hypointense structures that partially obstruct the endometrial cavity. Figure 2b shows a representation of the endometrium-dependent structure and its anatomical location.

Because of this finding we performed a magnetic resonance imaging (MRI) scan of the pelvis, which showed a polypoid endometrium-dependent structure with its base adjacent to the internal cervical os, it showed an approximate diameter of 1.4 cm. The signal was heterogeneous secondary to cystic-like interposed structures, as well as the presence of a 0.3 cm band-like structure at the base of the lesion. A more detailed description of the MRI can be found in Figure 2.

The patient’s gynecologist decided to perform curettage and excision of the lesion through hysteroscopy. It was carefully resected and sent to the pathology department. They reported endometrial fragments with morphological findings of a polyp, as well as smooth muscle fragments without relevant alterations.

## Discussion

An endometrial polyp is an abnormal growth containing glands, stroma and blood vessels projecting from the endometrium that occupies spaces small or large enough to fill the uterine cavity and are usually benign lesions. The majority of polyps are located in the fundus. They range in size from about 5 mm to as large as filling the whole uterine cavity. They can be found in all age groups, most common between 40 and 49 years. They can be pedunculated (if they have a large flat base) or sessile (absence of a stalk). The endometrium varies from normal cycling endometrium to simple or complex hyperplasia in the presence of endometrial polyps, and rarely endometrial cancer can be found. Endometrial polyps have been implicated in 50% of cases of abnormal uterine bleeding and 35% of infertility [2,3].

The pathogenesis and natural history of endometrial polyps are not very clear. They are believed to be related to estrogen stimulation because of an increased concentration of estrogen receptors (ERs), predominantly ER-alpha in polyp glandular cells compared with normal endometrium, and a decreased expression of progesterone receptors (PRs). Endometrial polyps are common in the pre- and postmenopausal women; however, they usually do not cause obstruction of the whole endometrial cavity nor its lack of distention. This latter aspect is of great importance because malignancies are a cause of obstruction and lack of distention of the endometrial cavity during imaging studies such as SIS and HSG [1,2,4].

Imaging is best on the 10th day of the menstrual cycle, when the endometrium is thinnest, to minimize false-positive and false-negative results. TVUS has a reported sensitivity of 19%-96%, a specificity of 53%-100%, a positive predictive value of 75%-100%, and negative predictive value of 87%-97% to diagnose endometrial polyps. Colour-flow or power Doppler may improve the diagnostic capability of TVUS. Colour-flow Doppler may demonstrate the single feeding vessel typical of endometrial polyps. Power Doppler has been reported to increase sensitivity to around 97%, while specificity and the negative predictive value may be increased to 95% and 94%. In contrast to the other imaging methods, MRI has the advantage of determining the extension of an endometrial lesion, as well as the myometrium and adjacent pelvic structures. When using a T2-weighted sequence, endometrial polyps are seen as low-signal intracavitary masses surrounded by high-intensity endometrium and fluid. This technique is useful if neoplastic lesions are suspected, such as endometrial carcinoma. In our case, this was relevant to discard because of the uncommon presentation of the obstructive polyp and the lack of distention of the endometrial cavity [2,5,6].

Currently, a conservative approach is considered the best therapeutic option. Hysteroscopic polypectomy remains the gold standard for surgical treatment. Removal of the endometrial basalis at the endometrial polyp origin appears to prevent recurrence of further endometrial polyps. Large polyps (>20 mm in diameter) and those located at the fundus are better removed by the resectoscope. Sessile polyps are best removed with an electrosurgical loop, while, small polyps (<20 mm) may be excised using an operating hysteroscope under direct vision with a pair of scissors. Although the malignancy rate of endometrial lesions in premenopause and menopause is minimal, a curettage can reduce abnormal uterine bleeding with >70% of success rate [1,3,7].

Dilation and curettage, combined with the use of polypectomy forceps, used to be the standard method for investigating abnormalities. This ‘blind procedure’ has well-known limitations, such as missed excision in 50%-85% of the cases due to their mobility. The most common complications associated with this procedure are risks associated with anesthesia, haemorrhage or heavy bleeding, infection in the uterine or other pelvic organs, uterine perforation, among others [7].

Our case reflects the relevance of imaging methods to have a better understanding of structural abnormalities that could explain the patient’s complaints. HSG remains an effective method to detect polypoid endometrial lesions, which are seen as filling defects. The drawback of this method is the use of ionic radiation and patient's discomfort because of its invasive nature. This method is also not capable of determining if the filling defect originates from the endometrium or myometrium. Therefore, other modalities such as MRI are a good option to assess the extension of these types of lesions, as well as both ovaries and the pelvic cavity [2,4-6].

## Conclusions

Endometrial polyps are a common cause of abnormal uterine bleeding and infertility. Nonetheless, they rarely block the endometrial cavity completely. Because of the unusual presentation of our case, it was imperative to perform additional imaging studies to have a more precise diagnosis. With this article, we want to expand the body of literature on the diagnosis of this condition.
